# Interpretation of Serological Complement Biomarkers in Disease

**DOI:** 10.3389/fimmu.2018.02237

**Published:** 2018-10-24

**Authors:** Kristina N. Ekdahl, Barbro Persson, Camilla Mohlin, Kerstin Sandholm, Lillemor Skattum, Bo Nilsson

**Affiliations:** ^1^Rudbeck Laboratory C5:3, Department of Immunology, Genetics and Pathology, Uppsala University, Uppsala, Sweden; ^2^Centre of Biomaterials Chemistry, Linnaeus University, Kalmar, Sweden; ^3^Section of Microbiology, Immunology and Glycobiology, Department of Laboratory Medicine, Clinical Immunology and Transfusion Medicine, Lund University, Lund, Sweden

**Keywords:** complement, deficiency, activation products, functional test, complement regulatory drugs

## Abstract

Complement system aberrations have been identified as pathophysiological mechanisms in a number of diseases and pathological conditions either directly or indirectly. Examples of such conditions include infections, inflammation, autoimmune disease, as well as allogeneic and xenogenic transplantation. Both prospective and retrospective studies have demonstrated significant complement-related differences between patient groups and controls. However, due to the low degree of specificity and sensitivity of some of the assays used, it is not always possible to make predictions regarding the complement status of individual patients. Today, there are three main indications for determination of a patient's complement status: (1) complement deficiencies (acquired or inherited); (2) disorders with aberrant complement activation; and (3) C1 inhibitor deficiencies (acquired or inherited). An additional indication is to monitor patients on complement-regulating drugs, an indication which may be expected to increase in the near future since there is now a number of such drugs either under development, already in clinical trials or in clinical use. Available techniques to study complement include quantification of: (1) individual components; (2) activation products, (3) function, and (4) autoantibodies to complement proteins. In this review, we summarize the appropriate indications, techniques, and interpretations of basic serological complement analyses, exemplified by a number of clinical disorders.

## The complement system

### Activation of complement

**Figure 1:**
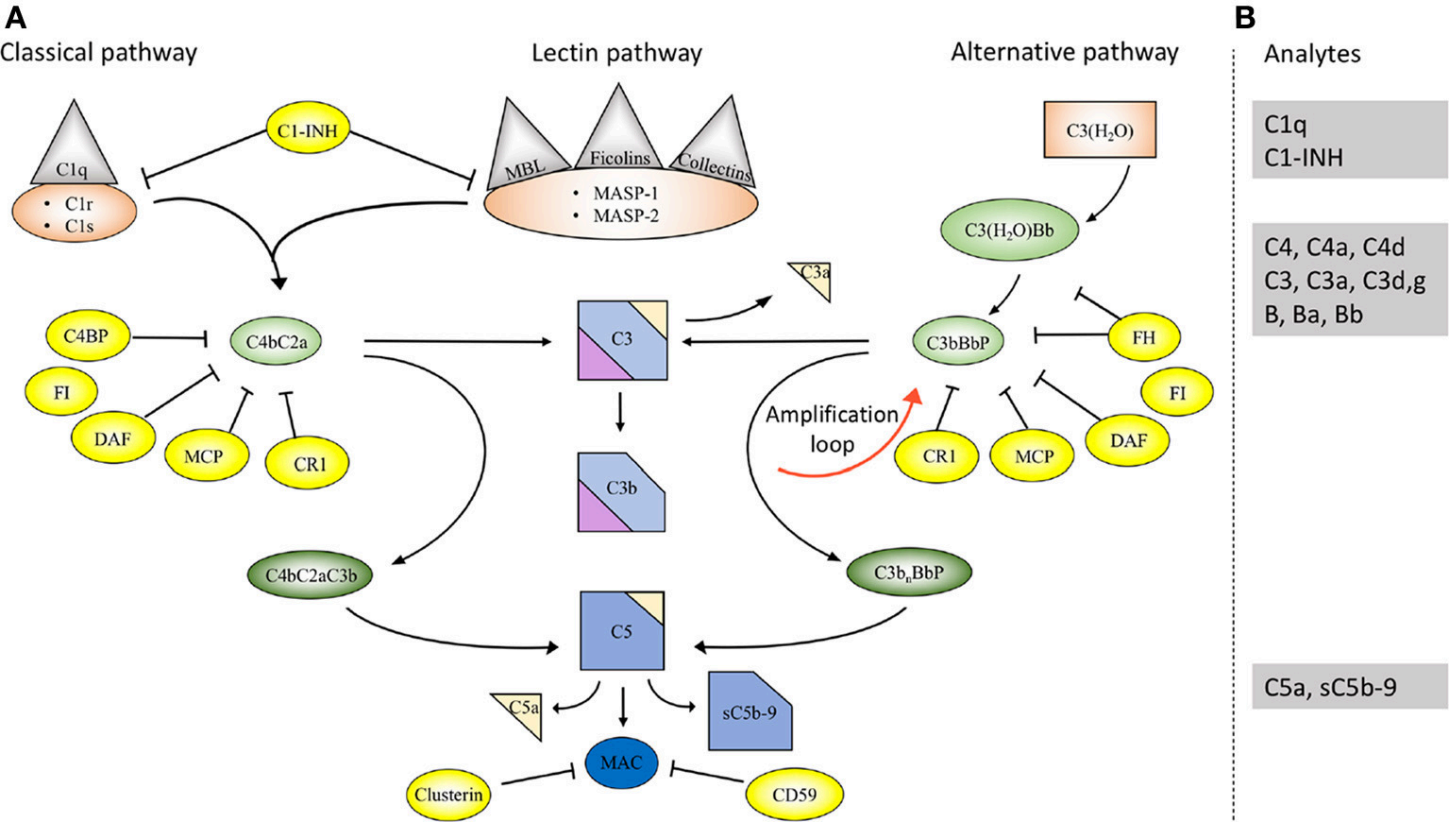
Overview of the complement system. **(A)** Activation and regulation of the complement system. The complement system can be activated by three different pathways: the classical (CP), lectin (LP), and alternative pathways (AP). Recognition molecules within the three pathways bind to structures present on pathogens, and then activate the serine proteases C1r and C1s of the CP and MASP-1, MASP-2 of the LP, respectively. These proteases initiate the assembly of the CP/LP C3 convertase (C4bC2a) while formation of the AP C3 convertase (C3bBbP) is initiated either by hydrolysis of C3 to C3(H_2_O) or by other mechanisms. The convertases cleave C3 to the opsonin C3b and the anaphylatoxin C3a. Subsequent action of the C5 convertases generates the anaphylatoxin C5a and initiates the assembly of the C5b-9 complex. Complement activation is under strict control of a number of membrane-bound and fluid phase inhibitors, the majority of which control the activity of the convertases. These include (but are not restricted to) C4b-binding protein (C4BP), decay acceleration factor (DAF), membrane cofactor protein (MCP), and complement receptor 1 (CR1), factor H (FH), and factor I (FI). In addition, formation of the C5b-9 complex is under control of CD59 and clusterin, while the CP and LP serine proteases are inhibited by C1- inhibitor (C1-INH). Color coding: recognition molecules: gray triangles; initiators serine proteases and C3(H_2_O): orange symbols; convertases: green ovals; inhibitors: bright yellow ovals; anaphylatoxins: dim yellow triangles. **(B)** Selected analytes commonly used clinically to assess complement function and activation. C1q, C1-INH, C4, C3 factor B (B) and their activation fragments, as well as C5a and sC5b-9.

The complement system comprises approximately 50 proteins that are found in the fluid phase of the blood or bound to cells where they function as receptors or regulators of complement activation (Figure 1A). The system is organized in three activation pathways: the lectin pathway (LP), the classical pathway (CP), and the alternative pathway (AP), each with different recognition molecules. Complement activation leads to the formation of two proteolytic enzyme complexes, convertases which have C3, the central and most abundant complement component as their common substrate.

The LP is activated when one of several recognition molecules, mannan-binding lectin (MBL), collectins, or ficolins bind to carbohydrates, e.g., on a pathogen surface, and often on polymers. The CP is activated when C1q binds to IgM or IgG, which may be in the form of immune complexes or bound in an altered conformation to artificial surfaces, such as in medical devices. In addition, the CP can also become activated by pentraxins, e.g., C-reactive protein (CRP), and a variety of negatively charged molecules that includes DNA, LPS and heparin. This activation leads to activation of fluid phase proteolytic enzymes: mannan associated serine proteases (MASP)−1 and MASP-2 within the LP and C1r and C1s within the CP. These proteases mediate formation of the CP/LP C3 convertase C4bC2a.

Activation of the AP is accomplished by conformationally altered C3, either as a result of tick-over to C3b or C3(H_2_O) or by its binding to surfaces, but AP activation can also be facilitated through the binding of properdin to damage-associated molecular patterns (DAMPs) on pathogens (1).

Through these processes of activation, the formation of the AP C3 convertase, C3bBbP, is induced. The labile C3 convertases cleave C3 into the anaphylatoxin C3a and the larger C3b fragment. In the presence of an acceptor surface, e.g., a pathogen or antigen-antibody complex, C3b can form a covalent bond to amino acid or sugar residues. Then C3b can be cleaved in three steps by the plasma protease factor I, in cooperation with one of several co-factors. The two first cleavages generate iC3b, which promotes phagocytosis via interaction with different complement receptors (CR)-1 (CD35), CR3 (CD11b/CD18), CR4 (CD11c/CD18), and/or CRIg (complement receptor of the immunoglobulin family). The third factor I mediated cleavage separates the molecule into the target bound C3d,g fragment which is a ligand for CR2 (CD21), and C3c which is released from the activating surface. The same digestion also takes place in the fluid phase indicating that complement activation *in vivo* or *in vitro* can be monitored by measuring C3d,g, iC3b or C3a.

In addition to being a trigger of complement activation the AP also provides a potent amplification loop. Since each deposited C3b residue (regardless of the nature of the initial activation trigger) is the potential nucleus of a novel C3bBb C3 convertase, it has the potential capacity to activate numerous other C3 molecules. Deposition of additional C3b molecules to or in the vicinity of either of the C3 convertases alters their enzymatic specificity from C3 to C5. Cleavage of C5 yields the anaphylatoxin C5a and initiates the generation of the terminal pathway (TP) where the end product is the terminal complement complex, C5b-9, which may remain in the plasma as soluble C5b-9 (sC5b-9) or be inserted in the cell membrane as membrane attack complex (MAC). MAC may induce cell lysis (primarily in non-nucleated cells) and gram-negative bacteria or inflammation and upregulation of tissue factor, e.g., on endothelial cells, at sub-lytic concentrations (2, 3).

The anaphylatoxins C3a and C5a bind to their receptors C3aR and C5aRs, expressed on phagocytes: polymorphonuclear cells (PMNs), and monocytes, thereby attracting and activating them, thus further fuelling the inflammation.

### Regulation of complement

A number of regulators protect surfaces of autologous cells against complement attack. These regulators include (but are not restricted to) cell-bound molecules, such as CR1, decay acceleration factor (DAF; CD55), and membrane cofactor protein (MCP; CD46), all of which inactivate the C3 convertases in different ways. Additional regulators, C4b-binding protein (C4BP, which regulates the CP/LP convertase) and factor H (the main regulator of the AP), found in the plasma are recruited via glycoseaminoglycans and/or deposited C3 fragments to the cell surface, thus providing further down-regulation of complement.

Regulation at the level of the TP is accomplished by cell bound CD59, and clusterin and vitronectin in the fluid phase, which all inhibit MAC formation and its insertion into the membrane of autologous cells. Furthermore, C1 inhibitor (C1-INH) inhibits the proteases generated within the CP and LP; C1r/C1s and MASP-1/MASP-2, respectively, (Figure 1A). However, C1-INH is not specific for complement system-associated serine proteases but also inhibits proteases generated by the activation of the contact system like Factor (F)XIIa, FXIa, and kallikrein.

### Pathology of complement

**Figure 2:**
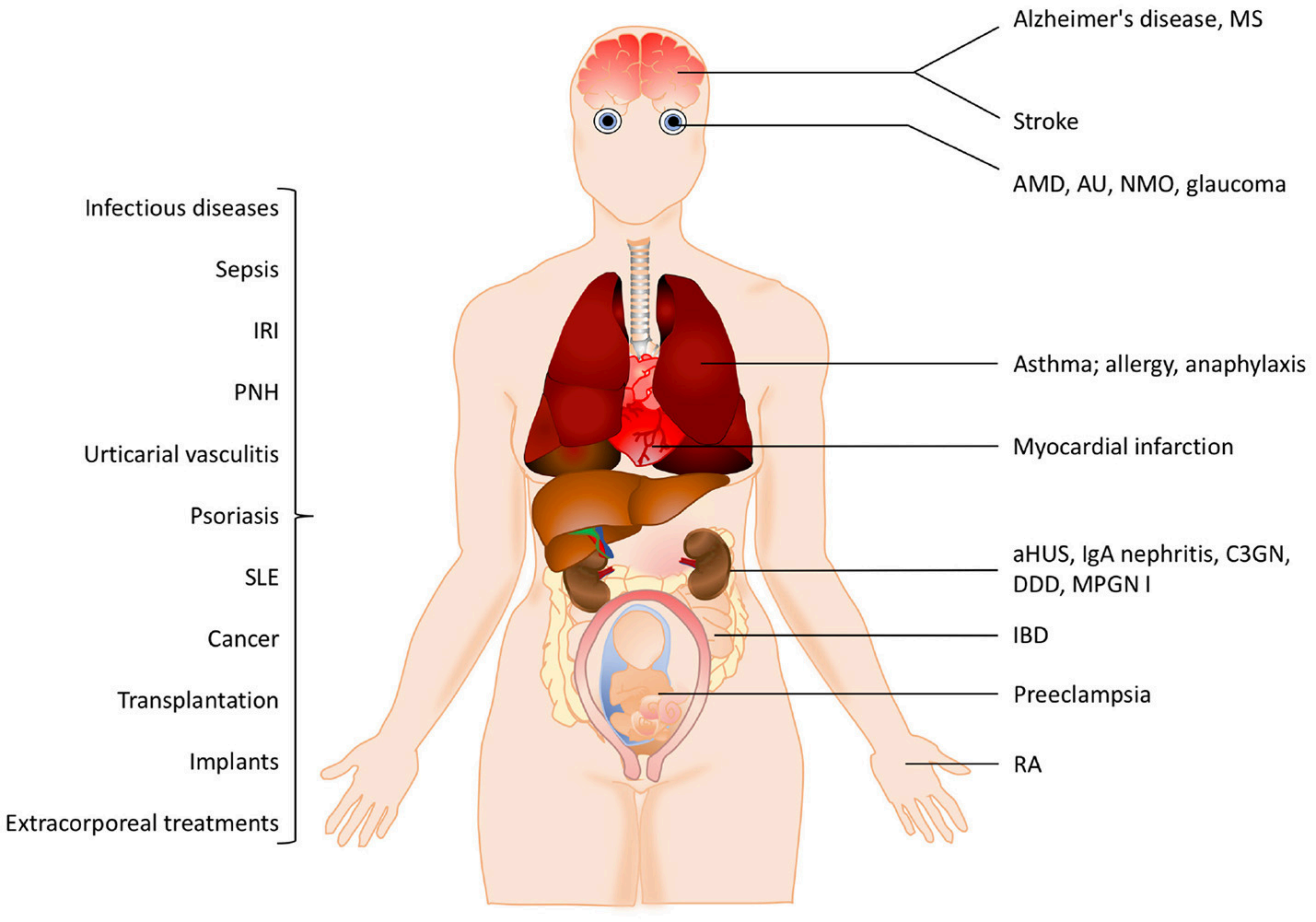
Examples of pathological conditions involving the complement system. The pathogenesis of many inflammatory diseases includes excessive or uncontrolled complement activation. Some of these pathological conditions are organ-specific while others are systemic. In addition, different treatment modalities such as transplantation, implants or extracorporeal treatments also trigger complement activation. See the text for details. IRI, ischemia reperfusion injury, PNH, paroxysmal nocturnal hemoglobinuria; SLE, systemic lupus erythematosus; MS, multiple sclerosis, AMD, age-related macular degeneration; AU, autoimmune uveitis; NMO, neuromyelitis optica; aHUS, atypical haemolytic uremic syndrome; C3GN, C3 glomerulonephritis; DDD, dense deposit disease; MPGN, membranoproliferative glomerulonephritis; IBD, irritable bowel disease; RA, rheumatoid arthritis.

The pathogenesis of many inflammatory diseases includes different complement deficiencies as well as excessive complement activation. Complement is engaged in a number of diseases exemplified in Figure 2. The pathologic effect may be caused either by an increased and persistent activation or an altered expression or function of various complement inhibitors resulting in defective control. Systemic lupus erythematosus (SLE), myasthenia gravis and other autoimmune disorders are examples of the former, where the presence of soluble or solid-phase antibody-antigen complexes induce excessive complement activation. C3 glomerulopathy (C3G), paroxysmal nocturnal haemoglobinuria (PNH), and atypical haemolytic uremic syndrome (aHUS), are diseases which are associated with insufficient complement inhibition/regulation, e.g., as discussed in (4, 5) and quoted in the references.

In many cases the complement activation is a part of reactions resulting from activation of all cascade systems of blood, and under conditions such as ischemia/reperfusion injury (IRI), there is a combination of excessive activation and insufficient control. IRI can occur under many pathological conditions but also during medical treatments. Cardiac infarction and stroke are associated with ischemia followed by reperfusion of an organ or blood vessel. Ischemia which is often complicated with IRI can also occur after transplantation (both allogeneic and xenogeneic) as well as during cardiovascular surgery facilitated by cardiopulmonary bypass. In IRI, excessive complement activation in combination with insufficient complement regulation play important roles and the resulting damage appears to be associated with all three pathways of complement. This complement activation leads to an inflammatory response which consists of generation of anaphylatoxins and other mediators which collectively induce activation of endothelial cells and phagocytes resulting in recrutiment and extravasation of PMNs, as described in (6) and cited references.

Despite the clear involvement of complement in a large number of conditions, there are only a limited number of diseases where serological complement biomarkers have been established as differential markers of disease. In the majority of other conditions, the biomarkers are able to distinguish between patients and normal individuals at group level. However, these markers can often be used to follow individual patients if the baseline values are known or can be anticipated e.g. as in trauma and shock (7), sepsis (8), in neurological diseases such as myasthenia gravis (9), in ophthalmic diseases such as age-related macular degeneration, (AMD) (10), bullous pemphigoid (11), antineutrophil cytoplasmic antibody (ANCA)-associated vasculitis (12) etc. They can also be used for research purposes.

This review will describe the indications and specific methods that are used to determine the complement status of a patient and how the results of these assays are interpreted.

## Analytical methods

### Activation *in vivo* vs. activation *in vitro*

**Figure 3:**
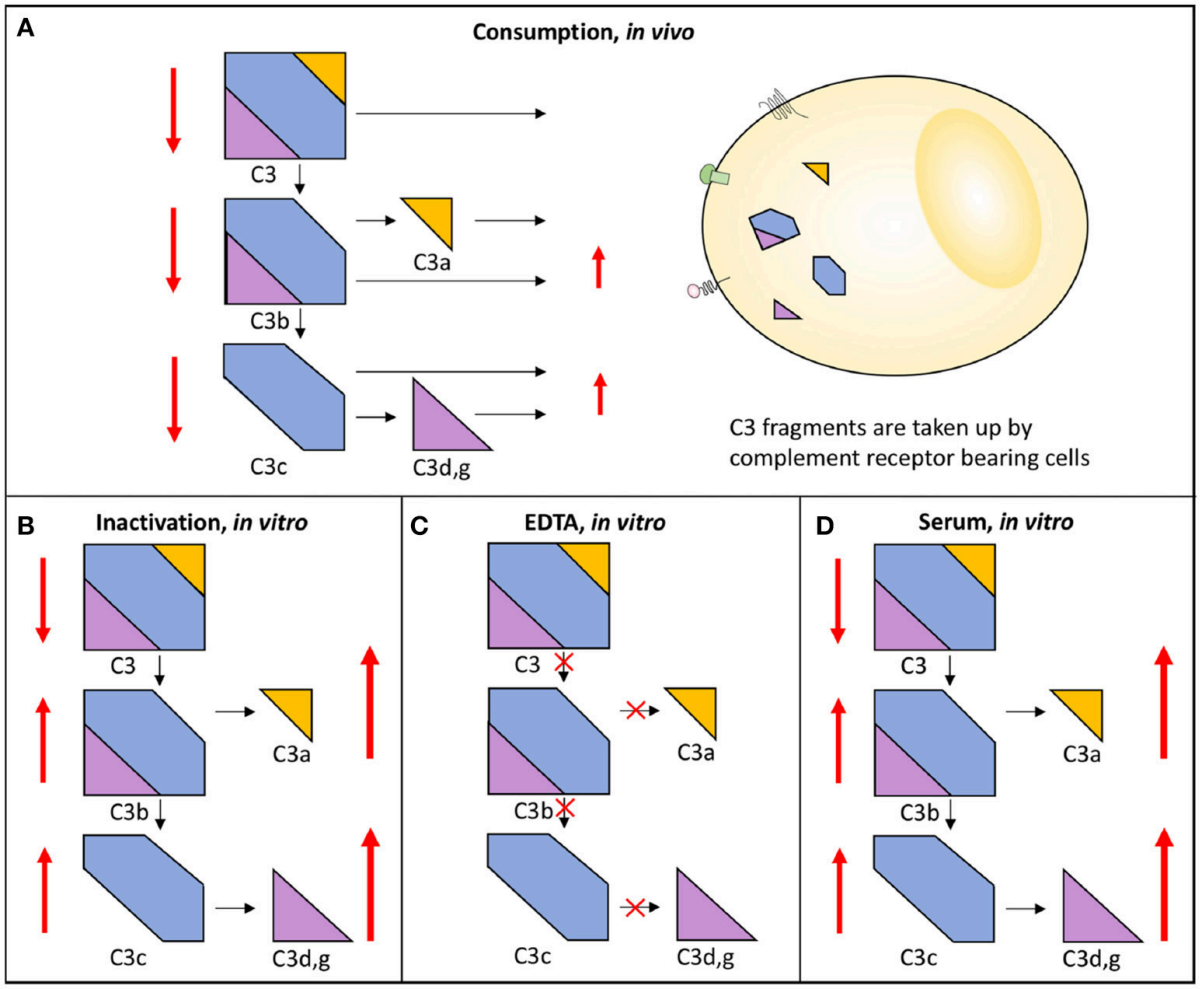
Activation and consumption of complement *in vivo* and *in vitro*. Activation of C3 yields the activation products C3a, C3b/iC3b, C3d,g, and C3c (indicated by arrows). *In vivo*, a portion of the C3 fragments get eliminated by complement receptor-bearing cells **(A)**. The complement system can also be activated *in vitro* in serum **(D)** or in maltreated samples **(B)**, but in these cases the activation products remain in the fluid phase. In order to avoid complement activation *in vitro*, blood can be drawn in the presence of EDTA which inhibits all further activation is **(C)**. In each panel, the degree of C3 cleavage is indicated by the length of the arrows.

Complement system activation via different pathways in blood plasma is a feature of a large number of diseases. For example, in immune complex diseases, the CP and the TP components are mainly activated while in renal diseases the AP and the TP components are predominantly engaged. When a component is activated *in vivo* either by proteolytic cleavage and/or by induced conformational changes triggered by protein-protein interactions, the component is taken up by receptors of e.g., leukocytes and Kupffer cells. This results in consumption of complement components. If a whole pathway (CP+TP or AP+TP) is activated, all components are consumed and the function is reduced along this pathway and systemic activation products will be moderately increased. Poor function via either the LP/CP or the AP will also affect the other pathway if the activation is strong enough since the components of the common TP will be consumed. If on the other hand the pathway (CP+TP, LP+TP or AP+TP) is activated *in vitro* all components are inactivated along this path and the function of this activation pathway is also reduced, but unlike the *in vivo* situation the activated proteins remain in the sample and are not consumed and the activation products will stay at high levels in the tube. If EDTA-plasma is prepared, any further activation of complement is stopped, since EDTA chelates Ca^2+^ and Mg^2+^ and thereby blocks the function of the C1 complex and the two C3 convertases, respectively. If, by contrast, serum is prepared, further activation of the sample *in vitro* is possible and it can be used for functional testing, e.g., using haemolytic assays (Figure 3) (13).

### Preanalytical factors

With this in mind, EDTA-plasma is suitable for analyses of individual components and for activation products, while serum is used for analysis of complement function. Serum can be replaced by plasma anticoagulated with the specific thrombin inhibitor lepirudin (i.e., recombinant hirudin) or any other thrombin or FXa inhibitor, which does not affect complement function (14). In order to maintain the function of the complement components and avoid further activation of individual components the samples must be kept cold until they are frozen at −80°C, which should be done preferably within 120 but no longer than 240 min. It is important not to freeze the samples at −20°C, not even temporarily, since this creates a slow freezing rate and further activation/inactivation of individual components. During transportation dry ice must be used and the samples should be transferred directly from the to 80°C freezer to the dry ice (13).

### Analysis of complement in plasma/serum

#### Quantification of individual complement components

Different types of immunoassays, most commonly immunoprecipitation assays, are used to determine the concentration of individual complement components. Previously, rocket immuno electrophoresis (RIE), radial immunoprecipitation or enzyme immune assays (EIAs) were most common, but have today to a great extent been replaced by nephelometry and turbidimetry. These techniques utilize polyclonal antibodies against the analyte, e.g., C1-INH, C4, C3, or factor B or activation fragments of these proteins (Figure 1B). These antibodies are added in excess to the sample and bind to their target, forming antigen-antibody complexes. Detection is performed by passing a light beam though the sample and which will be dispersed or absorbed by the formed immune complexes.

All techniques which use polyclonal antibodies for detection are relatively robust regarding the effect of preanalytical factors such as proteolytic cleavage or denaturation of the target protein induced during suboptimal sample handling. However, it is important to be aware that when polyclonal antibodies which are raised against C3c are being used, these assays will detect all forms of C3 which contain the C3c moiety, i.e, intact, non-activated C3 and its activated proteolytic fragments C3b, iC3b, and C3c. In analogy, anti-C4c antibodies will detect the corresponding forms of C4. Consequently, this type of assay is useful to determine the *in vivo* concentration of the protein (i.e., to monitor consumption or deficiency) but gives no information of the activation state or conformation of the protein.

More recently, multiplex assays for complement components have been developed and are now commercially available. The advantage of such assays is that they enable the simultaneous determination of several components, thereby saving both time and sample volume. So far, the analytes in the available kits are restricted to components with fairly high plasma concentrations, and to our knowledge, no LP-specific panels are yet on the market.

#### Quantification of activation products

The sequential proteolytic cleavage which occurs during complement activation generates activation products with different properties than those of the non-activated zymogen molecules (Figure 1B). In general, two principles are used in assays designed to determine the degree of complement activation: one is to use monoclonal antibodies (mAbs) which detect amino acid sequences that are inaccessible in the native zymogen molecule but become exposed when the protein is activated (i.e., neo-epitopes). Most commercially available assays for C3a, C3b/iC3b/C3c, C4a, C4b, C4d, Ba, Bb, C5a, and sC5b-9 are based on neo-epitope mAbs. The other option is to use polyclonal antibodies but this often requires fractionation of zymogen molecules and activation products according to size. One example is C3d,g which is detected by EIA or nephelometry/turbidimetry, but since the polyclonal antibodies (in this case raised against C3d,g) also recognize intact C3, C3b, and iC3b in addition to C3d,g, these larger molecules must be removed by precipitation before analysis (15).

*In vivo* there is a continuous physiological turnover of C3 which leads to generation of activation fragments including C3a and C3d,g. Consequently, in order to monitor ongoing complement activation it is mandatory to determine a ratio of C3a or C3d,g level to the total level of C3 (C3a or C3d,g/C3), e.g., during an exacerbation in SLE (15). Furthermore, in obese individuals, the levels of a number of complement components, including C3, are greatly increased, resulting in corresponding higher levels of C3a; this problem further underscores the importance of calculating a ratio as a measure of relative activation (16).

Formation of the lytic C5b-9 (MAC) complex is the last step of the complement cascade which causes cell damage or lysis as a result of its insertion into the cell membrane, or endothelial cell activation at sub-lytic concentrations (2, 3). Complement activation of the TP can be monitored by quantification of sC5b-9 in the fluid phase, with an EIA which uses a mAb specific for a neo-epitope in C9 for capture. The epitope for this mAb is exposed in conformationally changed complex-bound C9 but not in intact C9. After capture, the sC5b-9 complexes can be detected by using polyclonal antibodies against another protein present in the same macromolecular complex, e.g., C5 or C6 (17).

Most, if not all, complement activation markers can rapidly be produced by complement activation *in vitro*. Consequently, it is of utmost importance that samples intended for detection of complement activation are collected and handled properly. In particular, there is a great risk that C3a will be generated *in vitro* if samples are improperly handled (14). It should also be taken into account that different C3 activation products vary greatly with regard to their *in vivo* half-life: approximately 0.5 h for C3a (18) and 4 h for C3d,g (19). Since C3d,g is a more robust marker, it is more suitable for diagnostic use while the generation of C3a is the more common analysis in experimental settings (20).

#### Quantification of complement function

In order to maintain full function in an individual complement activation pathway it is necessary for each of the participating proteins to be active, i.e., a deficiency in one individual protein will stop the activity of the entire cascade. Functional tests, in particular different haemolytic assays that monitor a whole activation pathway from the recognition phase to MAC-formation (= lysis) can be used to detect both deficiencies in individual component as well as depression in complement function caused by consumption of intact complement components.

**Figure 4:**
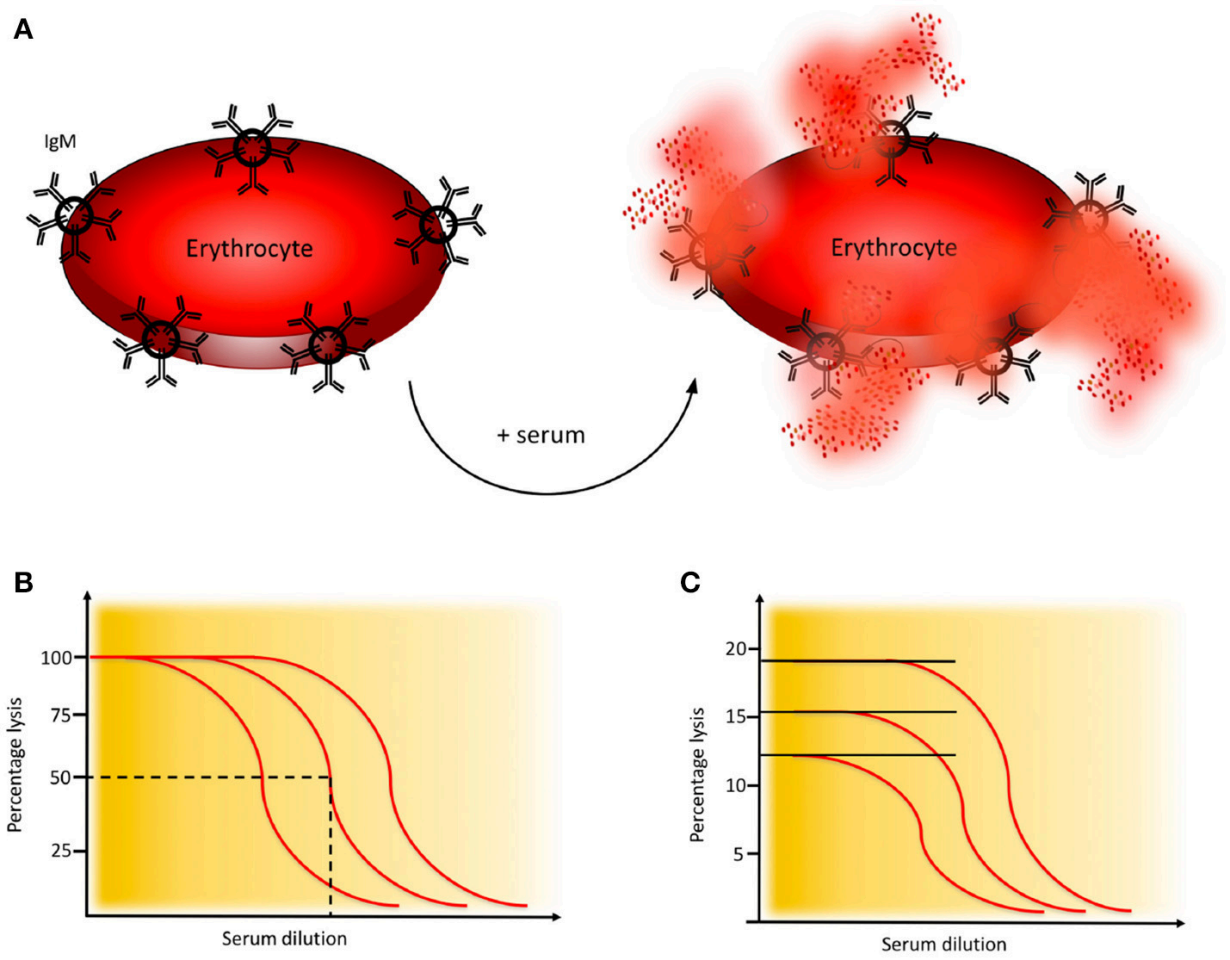
Haemolytic assay for detection of complement classical pathway (CP) function. **(A)** CP haemolytic assay. Sheep erythrocytes coated with IgM antibodies are incubated with patient serum. The C1 complex binds and initiates formation of the CP convertase, leading to activation of C3, assembly of the C5b-9 complex, and subsequent erythrocyte lysis. **(B)** CH50 assay. Titration of the amount of serum needed to lyse 50% of a specified limited and fixed quantity of cells in the CH50 assay. The curves show three individuals with different levels of complement function. **(C)** One tube CP assay. Since the activity of complement is proportional to the quantity of cells that are lysed, this assay is performed in an excess of erythrocytes. The curves show three individuals with different levels of complement function. Assays for alternative pathway (AP) activation function similarly except that uncoated rabbit or guinea pig erythrocytes which spontaneously activate the AP, are used.

Activation of the CP of complement is monitored in haemolytic assays employing sheep erythrocytes coated with rabbit antibodies, preferably purified IgM (or mixed with IgG) to the Forssman antigen. Patient serum is added, C1q binds to the immunoglobulins which initiates formation of the CP C3 convertase and subsequent activation leads to assembly of the MAC which results in lysis of the erythrocytes, Figure 4A (21).

Activation of the AP of complement is monitored in haemolytic assays employing rabbit or guinea pig erythrocytes, which are spontaneous and potent activators of human AP. EGTA which chelates Ca^2+^ and thereby inhibits activation via the CP and LP, is added to the patient serum prior to incubation. Under these conditions the AP C3 convertase is formed on the target erythrocytes, leading to C3 activation and subsequent lysis (22).

A variety of haemolytic assays have been developed using different serum dilutions and amounts of erythrocytes. In the original haemolytic assays called CH50 and AH50, a specified limiting amount of erythrocytes are incubated with serum in serial dilution to determine the dilution of serum needed to lyse 50% of the cells during a certain time interval (Figure 4B) (21, 22). In samples with low function it is often necessary to repeat the analysis with additional dilution steps.

Unlike in the CH50 and AH50 assays, where incubation of erythrocytes and serum takes place in the fluid phase, an alternative approach is to cast the target erythrocytes in an agarose gel. The patient serum is then added into wells punched in the agarose and diffuses in the gel causing cell lysis. This haemolysis-in-gel technique is quick and very useful to screen for complement deficiencies but does not enable quantification (23).

Instead of using erythrocytes, which may cause problems due to individual variation of the animals that have donated the blood, systems using artificial liposomes have been developed. Assays which are commercially available is performed in a CH50-like way (24).

An alternative to the CH50 and AH50 assays is the considerably less laborious, and much quicker one-tube assay. The sample is here incubated in one tube for 20 min. These assays give similar results as CH50 and AH50 (21, 25) and are based on the fact that the “dose” of complement is proportional to the number of cells lysed and the assay is therefore performed in an excess of erythrocytes (Figure 4C).

**Figure 5:**
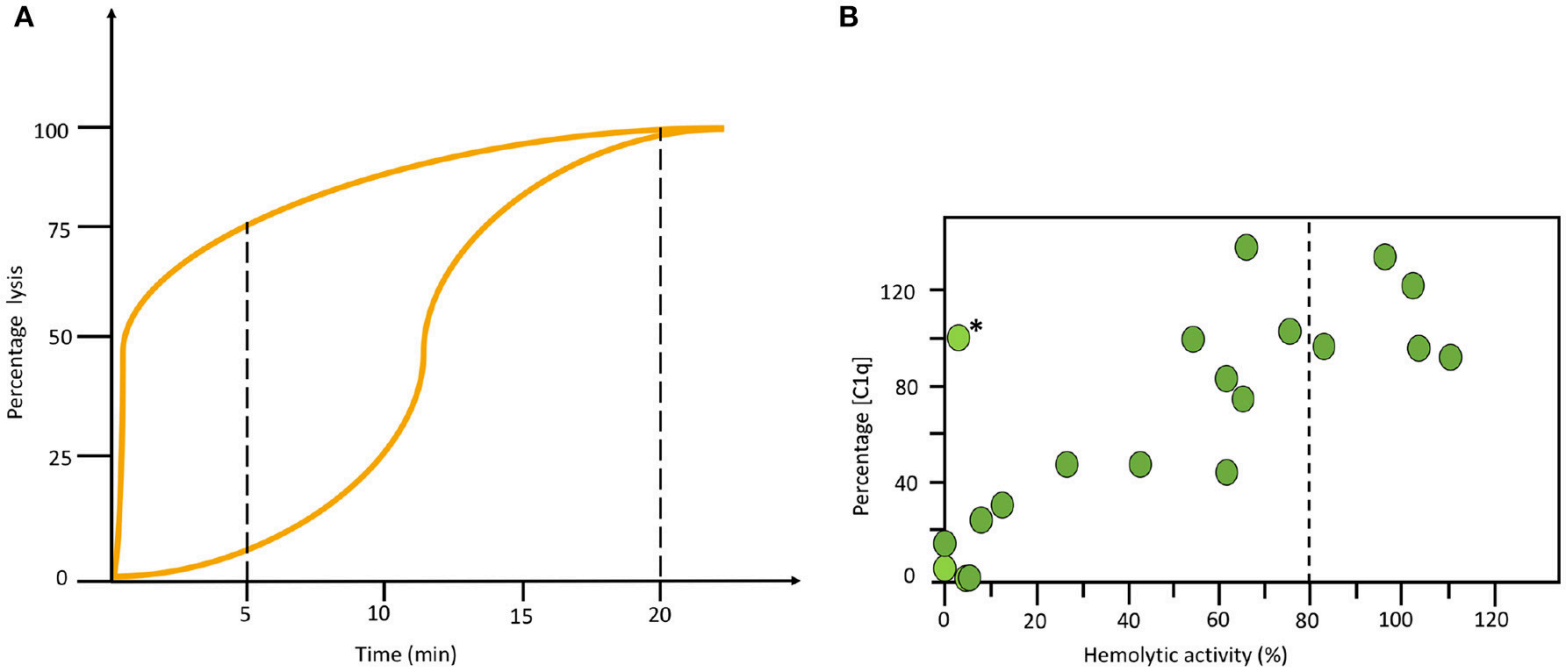
Examples of specialized functional assays. **(A)** Time course alternative pathway (AP) haemolysis test. The left curve was obtained using serum form a complement sufficient individual and the right curve using serum form a properdin deficient patient. Since the curves merge at the same level after 20 min in the one tube assay it is necessary to also incubate for shorter time to locate this deficiency. **(B)** Correlation between functional test and individual analytes. Combination of haemolytic function via the classical pathway (CP) and an individual analyte (C1q) show a correlation in this material of systemic lupus erythematosus (SLE) patients. The exception, marked with (^*^) is a patient with total C2 deficiency. **(B)** reproduced from (15) with permission from the publisher.

All haemolytic assays have problems to detect properdin deficiencies. Normally they give intermediate to normal values, never low function as is seen in other deficiencies. Therefore, special arrangements need to be made. One way is to make a kinetic analysis of the sample in the AP haemolytic assay. In the example shown in Figure 5A it is seen that the curves for a properdin deficient patient and a healthy control merge at the same level after 20 min in the one-tube assay. Therefore, it is necessary to also incubate for shorter time to detect this deficiency (Figure 5A). Alternatively, the concentration of properdin is determined separately.

**Figure 6:**
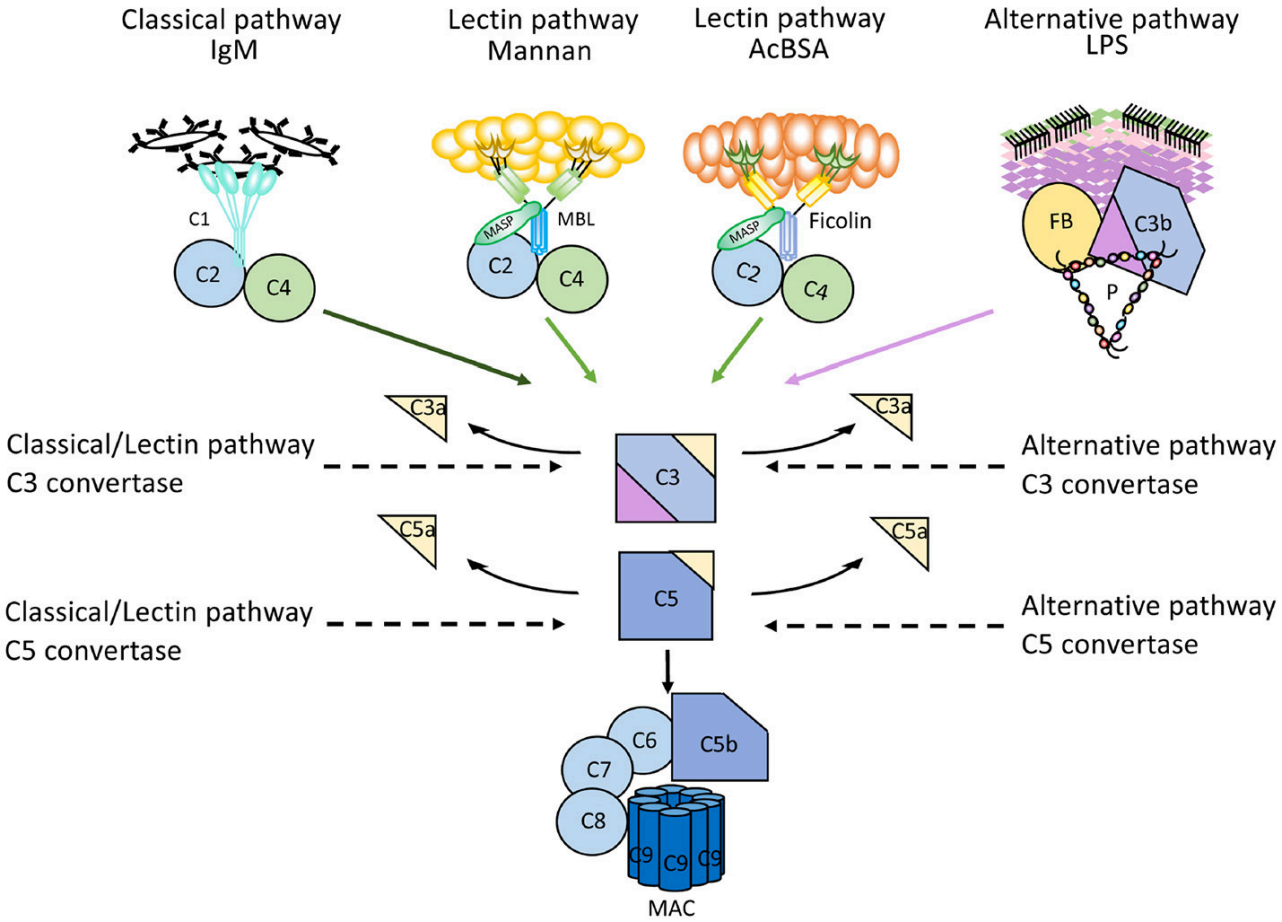
Mechanism of action of microtitre plate based complement activation assays. Schematic representation of the mechanisms for commercially available enzyme immune assays (EIA) to monitor complement activation via the three different pathways. Wells of microtiter plates are coated with ligands for the recognition molecules within each pathway. For each assay, serum supplemented with inhibitors for the other two pathways is incubated. After complement activation, the readout for all assays is formation of the terminal complement complex C5b-9.

More recently, a method has been reported that makes use of parallel EIAs to quantify the function of the three activation pathways of complement (26). Target molecules for each pathway are coated on wells of microtitre plates; IgM for the CP, mannan or acetylated bovine serum albumin for the LP, and LPS for the AP. Patient serum is incubated in the wells in the presence of additions which enable specific activation of only one pathway at the time, since the activity of the other pathways are inhibited. The readout for each EIA is formation of C5b-9 which is detected by a mAb specific for a neo-epitope in C9 which is exposed in complex-bound but not in native C9 (17). These assays are commercially available (Figure 6).

The techniques described here are valuable to identify complement deficiencies and for the haemolytic assays (except the haemolysis-in-gel) also to monitor levels in complement function, for example in patients with SLE during exacerbations. A suspected deficiency can be confirmed by determination of the protein, using relevant assays as described above. Furthermore, since most plasma complement components are commercially available, it is possible to verify the deficiency by reconstituting the patient sample with the protein in question and then repeat the functional assay, where the activity should be normalized. i.e., by combining these techniques it is possible to distinguish between functional deficiency and lack of a single complement component (13).

## Examples of indications for complement diagnostics and the interpretation of complement status

The complement status of a patient cannot be determined using only one assay. In order to get a complete status, assays from all four categories of analyses have to be used (Table 1). Two major basic indications exist: identification of complement component deficiencies and monitoring of complement activation. In order to screen for complement deficiencies functional assays (haemolytic or EIA) are used. Here, both CP and AP assays are compulsory. For the LP, a commercial EIA to monitor activation via MBL exists, but it only covers MBL, MASP-1 and MASP-2. Also, C9 deficiencies may be missed depending on the erythrocytes used. For monitoring of the degree of complement activation a minimum set up is to use a functional assay triggered via the CP, C3 and an assay for activation products (iC3b, Ba, C3d,g etc.). Examples of add-on assays are the concentrations of, e.g., C1q, C4 and factor B, and a functional AP assay, e.g., a haemolytic assay (Figure 5B).

**Table 1:** Main indications for complement diagnostics.

DEFICIENCIES OF COMPLEMENT COMPONENTS
Increased susceptibility to bacterial infections
Repeated severe (invasive) bacterial infections
Meningococcal disease
Autoimmune disease (SLE and related diseases)
HAE (C1-INH deficiency)
AAE (C1-INH deficiency)
DISEASE ACTIVITY AND DIFFERENTIAL DIAGNOSIS
SLE
Antiphospholipid syndrome
Urticarial vasculitis
Cryoglobulinemia
Various (RA, GPA, Henoch Schönlein)
C3 glomerulopathy and other types of glomerulonephritis
Thrombotic microangiopathies (aHUS)
Transplantation (AMR)
ASSESSMENT OF THERAPEUTIC EFFECTS
C1-INH
Eculizumab

*SLE, systemic lupus erythematosus; HAE, hereditary angioedema,; AAE, acquired angioedema; C1-INH, C1 inhibitor; RA, rheumatoid arthritis; GPA, granulomatous polyangiitis; antibody mediated rejection; aHUS, atypical haemolytic uremic syndrome*.

### Inherited and acquired complement component deficiency

#### Complement factor deficiencies (general)

In general, complement deficiencies are rare, but when diagnosed, they are generally associated with recurrent bacterial infections (this applies to all activation pathways) (Table 2).

**Table 2:** Hereditary complement deficiencies.

LECTIN PATHWAY
MBL
Ficolins
MASPs 1-3
MBL, MASPs: increased susceptibility to bacterial, viral and protozoan infections MASPs1/3: 3MC syndrome
CLASSICAL PATHWAY
C1q, C1r, C1s
C4
C2
Bacterial infections, SLE
C2 deficiency: cardiovascular disease
ALTERNATIVE PATHWAY
Factor D
Factor B
C3
Properdin
Factor H
Factor I
Bacterial infections, e.g., *Neisseria, Haemophilus, Pneumococci*
TERMINAL PATHWAY
C5
C6
C7
C8
(C9)
Bacterial infections, e.g., *Neisseria*

*MBL, mannan-binding lectin; MASPs, MBL associated serine proteases; 3MC syndrome Malpuech-Michels-Mingarelli-Carnevale syndrome; SLE, systemic lupus erythematosus*.

In addition, individuals deficient in MBL or MASPs of the LP are also susceptible to viral and protozoan infections and deficiencies in CP components are generally associated with an increased incidence of SLE or SLE-like disease. Most susceptible to autoimmune disease are patients with C1q deficiency while individuals with C2 deficiency are less predisposed to this type of diseases. C2 deficiency is the most common CP specific deficiency with a frequency of 1/20,000 (27, 28).

A deficiency is detected by functional assays and gives a very low to non-existing function via one (AP-, CP-, LP- specific deficiency) or all pathways (TP specific). In order to confirm that the functional defect is due to a specific deficiency, cryoglobulinemia has to be ruled out. Cryoglobulins can totally inactivate the CP+TP in serum (or lepuridin anticoagulated plasma) after the sample has been drawn. Also, severe consumption due to complement activation in vivo has to be excluded by measuring complement activation products, e.g., C3d,g. Identification of which component is lacking is established by performing measurements of individual factor concentration, by Western blotting, genetic screening etc. Confirmation of the deficiency (see above) can be done by reconstitution of the deficient serum with the purified identified complement component.

#### Monitoring of complement regulatory drugs

Currently, there are only two complement inhibitors available in the clinic: C1-INH and eculizumab. Purified or recombinant C1-INH inactivates the proteases generated by the CP and LP (C1r, C1s, MASP-1, and MASP-2) as well as FXIIa, FXIa and kallikrein of the contact system (29). Eculizumab is a humanized mAb that binds to C5, preventing its activation to the anaphylatoxin C5a and C5b which initiates C5b-9 formation. Treatment with eculizumab is approved for treatment of aHUS, PNH, and refractory myasthenia gravis, but it is also currently undergoing clinical trials for the prevention of antibody-mediated rejection (AMR) in allogeneic kidney transplantation (30). In addition to these two drugs, a large number of complement-modulatory compounds that act at different control points are under development for various indications. Examples of compounds which are in clinical trials include mAbs against C1s (31), which inhibit the CP, the peptide CP40 of the compstatin family, which blocks C3 activation by the convertases of all three pathways (32), and APT070 (33), which inhibits the C3 convertases thereby blocking down-stream complement activation. In this field, there is a pressing need to monitor the complement status in all patients receiving treatment with complement-regulatory drugs, a need that is only expected to increase in the future. In most cases monitoring can be achieved using CP and AP functional tests (either EIA or haemolytic), since an acquired deficiency of specific complement components is created. If the inhibitor is an antibody, direct binding assays to the specific antigen can supplement these assays. A comprehensive overview of the field of therapeutic complement inhibition is found in (34).

### Disorders with complement activation (Table 3)

#### SLE, antiphospholipid syndrome and urticarial vasculitis

**Figure 7:**
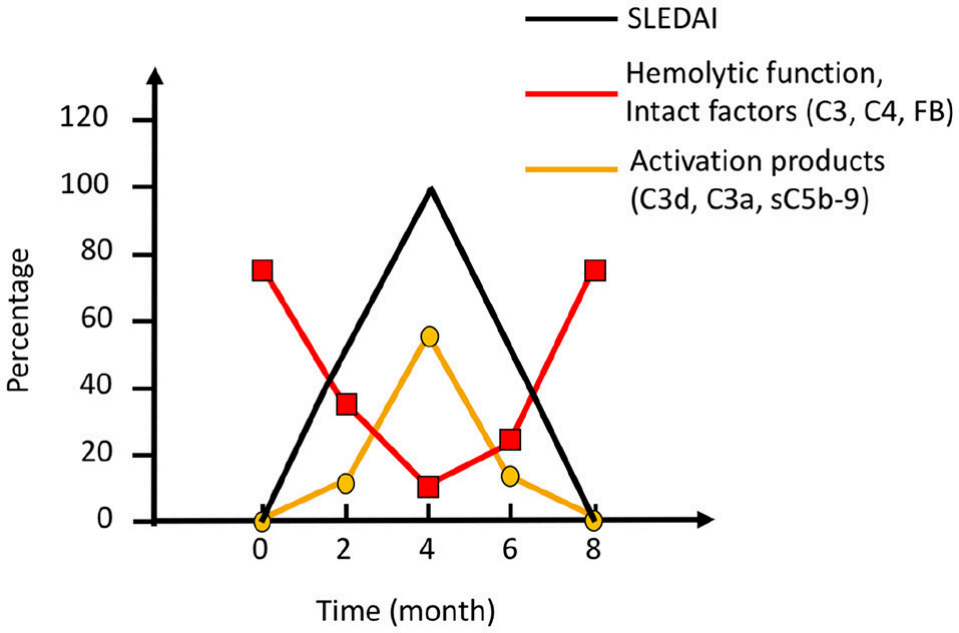
Complement activation during an SLE (systemic lupus erythematosus) excacerbation. Clinically, the magnitude of an excacerbation of (SLE) is quantified as SLE disease index (SLEDAI; black line). During excacerbation, intact complement components C3, C4, factor B (FB), and other components are consumed, which results in a decline in haemolytic function by the classical pathway (CP) (red line). Complement activation markers e.g., C3d,g, C3a, and sC5b-9 (orange line), peak concomitantly with the SLEDAI. Figure adapted from (13).

SLE, antiphospholipid syndrome and urticarial vasculitis are autoimmune immune complex diseases (35, 36). Other members of this group include rheumatoid arthritis with vasculitis, and cryoglobulinemia, as well as very rare cases of Henoch Schönlein disease and granulomatous polyangitiis (GPA) (37, 38). Complement analyses, in particular determination of CP function and analysis of components within the CP: C1q, C3, and C4 (C2 in some laboratories) are useful markers to monitor disease activity and for differential diagnosis (Figure 7). Furthermore, the detection of autoantibodies against C1q and C3 can be used to verify diagnosis (39–41). Hypocomplementemic urticarial vasculitis syndrome (HUVS) features anti-C1q antibodies with distinctive specificity as well as severe complement consumption via the CP (36, 42).

**Table 3:** Complement pathology.

**Analyses**	**CP function (%)**	**AP function (%)**	**C1-INH conc** **(g/L)**	**C1-INH funct** **(%)**	**C1q conc** **(g/L)**	**C4 conc** **(g/L)**	**C3 conc** **(g/L)**	**C3d conc** **(mg/L)**	**C3d/C3**	**sC5b-9 (μg/L)**
Reference interval	80–120	50–150	0.13–0.29	72–129	0.07–0.25	0.13–0.32	0.67–1.29	<5.3	<5.3	<25
Complement deficiencies CP, e.g., C2 deficiency	9	105	0.20	82	0.13	0.23	0.80	3.6	4.5	<25
Complement deficiencies AP, e.g., P deficiency	97	45	0.25	95	0.20	0.20	0.97	4.4	4.5	<25
Complement deficiencies TP, e.g., C6 deficiency	8	12	0.17	81	0.24	0.27	1.20	4.5	3.8	<25
SLE, urticarial vasculitis	10	109	0.17	79	0.06	0.08	0.65	6.3	9.7	75
Secondary phospholipid syndrome (SLE)	15	103	0.20	87	0.06	0.09	0.60	7.0	11.2	90
MPGN I	65	45	0.18	115	0.08	0.14	0.55	12	22	85
AMR	30	65	0.22	120	0.10	0.10	0.65	7	11	100
C3GN	15	10	0.26	122	0.22	0.15	0.10	18	180	200
PSGN	15	10	0.24	120	0.24	0.31	0.10	15	150	175
aHUS	95	105	0.19	103	0.20	0.21	0.75	6.5	8.7	75
HAE with low C1-INH conc	100	140	0.04	20	0.23	0.10	1.23	4.3	3.5	<25
HAE with dysfunct C1-INH	111	132	0.20	18	0.18	0.10	1.01	3.3	3.3	<25
AAE	93	94	0.03	11	0.05	0.09	0.80	3.9	4.9	<25
Eculizumab	5	10	0.23	79	0.25	0.26	1.17	4.2	3.6	<25

*CP, classical pathway; AP alternative pathway; C1-INH, C1 inhibitor; TP, termial pathway; SLE, systemic lupus erythematosus; MPGN, membranoproliferative glomerulonephritis; AMR, antibody mediated rejection; C3GN, C3 glomerulonephritis; aHUS, atypical haemolytic uremic syndrome; PSGN, post-streptococcal glomerulonephritis; HAE, hereditary angioedema, AAE, acquired angioedema*.

#### Antibody mediated rejection in transplantation (AMR)

AMR is the leading cause of long-term kidney graft loss (43, 44). The presence or formation of antibodies directed against the vascular endothelium in the graft is a major trigger of complement activation in transplantation, leading to microvascular inflammation and thrombosis followed by ischemia, apoptosis, or necrosis, and finally graft failure. AMR leads to a CP activation, which is presented in biopsies as C4d deposition. The majority of antibodies are either anti-blood group ABO antibodies (so called natural antibodies) or antibodies against HLA, due to previous immunization. In blood in a severe AMR the typical signs of a CP activation are seen with low CP function, low levels of C1q, C4, and C3, and increased activation products (e.g., C3d,g/C3, sC5b-9 etc.). In less severe AMR only raised levels of activation products can be seen.

#### C3 glomerulopathy (C3G)

C3 glomerulonephritis (C3GN) and dense deposit disease (DDD) are subsets of C3Gs that present a predominant C3 deposition in the glomerulus and is associated with C3NeF, other autoantibodies to complement, and in some cases mutations in complement protein genes (45). Since C3NeF binds to and stabilizes the AP C3 convertase, a profound C3 consumption occurs, that may lead to a functional C3 deficiency. The consumed C3 gives rise to C3d,g which is an indicator of substantial activation of C3. The level of factor B remains more or less unchanged. Since the stabilized convertase sometimes also cleaves C5, in some cases sC5b-9 can also be detected. Detection of C3NeF supports the diagnosis of C3G, in particular DDD. The severe C3 deficiency that results can, at least in theory increase the risk of bacterial infections.

#### Poststreptococcal glomerulonephritis (PSGN)

Another type of glomerulopathy associated with AP activation is the post-streptococcal glomerulonephritis (PSGN) that may occur during the rehabilitation period in individuals that have suffered from Group A streptococcal disease. In particular C3, but also C5, is consumed and sC5b-9 is generated for typically 6–10 weeks following the infection (45). Since the levels of C3 and C5 can be depressed and, as is true for C3G with/without C3NeF, there is a theoretical risk of other bacterial infections. The levels of C3d,g are elevated resulting in a high ratio of C3d,g to C3. PSGN is associated with a concomitant consumption of properdin, which is the major complement-related diagnostic difference between these diseases (45).

#### Atypical haemolytic uremic syndrome (aHUS) and other microangiopathies

aHUS is a disease that predominately appears in childhood and is characterized by thrombocytopenia, microangiopathic haemolytic anemia, and acute renal failure.

Affected cells in aHUS are endothelial cells, including those of the mesangium of the kidney as well as platelets and erythrocytes. aHUS is caused by uncontrolled complement activation due to combination of mutations of complement inhibitors, such as in factor H, but also factor I, factor H related proteins (FHR) 1, 3, 5, MCP, and thrombomodulin that impairs the function of these inhibitors (46). A deletion of the FHR-1/3 gene may lead to generation of anti-factor H antibodies which also is associated with aHUS. Gain of function mutations in C3 and factor B that lead to poorly controlled activation have also been reported (47, 48).

The most common cause of aHUS is mutations in the gene for factor H, and the majority of the mutations occur in the short consensus repeats (SCRs) 19 and 20 in the C-terminal of the molecule. These SCRs which interact with carbohydrates, e.g., heparan sulfate and sialic acid on the cell surface are important for binding factor H to the cell surface. Like in factor H dysfunction, other types of aHUS are also associated with AP activation, resulting in varying degrees of C3 consumption and the generation of C3d,g (and other C3 fragments) and sC5b-9. Factor H from aHUS patients may show different mobility compared to normal factor H when analyzed by SDS-PAGE, and western blotting. For a more precise diagnosis of AP components mutations, contact with a specialist laboratory is recommended.

In preeclampsia and other types of microangiopathy the levels of Bb and sC5b-9 are increased and various degrees of AP component consumption and low AP function can be seen (49).

### Inherited and acquired C1-INH deficiency

C1-INH deficiency is the cause of the rare disorders hereditary angioedema (HAE) and acquired angioedema (AAE), but HAE may also, in some cases be caused by mutations in the gene coding for FXII (50). The hereditary form, HAE, is heterozygous autosomal dominant, whereas the acquired form, AAE, mainly occurs in patients with underlying disease but can also be idiopathic. Since the cause of these diseases is an unregulated generation of bradykinin by the contact system, these are not primarily complement-system related diseases, but their diagnosis is based on complement analysis. Both HAE and AAE are associated with recurrent attacks of bradykinin-mediated, non-pitting, local angioedema that are not responsive to antihistamine or steroids.

There are different types of HAE, which can be distinguished only by laboratory analysis. Two types of C1-INH-related forms exist, one with low concentration and function of C1-INH (Type I), and one with normal concentration, but dysfunctional C1-INH (Type II). In contrast, HAE with normal C1-INH levels, which is not associated with low C1-INH function is a heterogenous group and therefore less characterized than the other types. In this group, certain patients have a gain of function form of FXII (Type III), due to a mutation in the coding gene leading to defective glycosylation (50).

Acquired deficiencies of C1-INH can occur in lymphoproliferative and autoimmune diseases as a result of formation of autoantibodies against C1-INH, or paraproteins e.g., M-components (51–53). C4 concentrations are typically low in both HAE and AAE (54). AAE occurs as the result of the hypercatabolism of C1-INH; in AAE, as opposed to HAE, the serum concentration of C1q is low in ~70% of patients.

## Autoantibodies to complement proteins (Table 4)

### Anti-C1q autoantibodies

Autoantibodies against C1q (anti-C1q) were first identified as low molecular weight C1q precipitins (55). Their immunoglobulin (Ig) nature was later confirmed, and the SLE-associated anti-C1q was shown to be specific for the collagenous region of the C1q molecule (56). Anti-C1q occurs in ~30% of unselected SLE patients but has a higher prevalence in lupus nephritis and is also associated with nephritis activity (57). In SLE, anti-C1q antibodies are often of the IgG2 subclass, but the reason for this selectivity is unknown. More than 95% of patients with HUVS are also positive for anti-C1q (58). However, anti-C1q antibodies are not specific for SLE and HUVS and may also be found in association with conditions such as primary glomerulonephritis and infectious diseases.

**Table 4:** Autoantibodies against complement components.

**Analyses**	**Anti-C1q^1^**	**Anti-C1q^2^**	**Anti-C1-INH**	**Anti-FH**	**C3NeF**	**C4NeF**
SLE, Sjögrens	+	–	(+)	–	–/(+)	–
Hypocomplementary urticarial vasculitis	+	+	–	–	–	–
C3GN	–/+	–	–	–/(+)	+	+
aHUS	–	–	–	+	–	–
HAE Type I, Type II	–	–	–	–	–	–
AAE	–	–	+	–	–	–

*Anti-C1q^1^, antibodies against native C1q; Anti-C1q^2^, antibodies against reduced chains of C1q; C3NeF, C3 nephritic factor; C4NeF, C4 nephritic factor; SLE, systemic lupus erythematosus; C3GN, C3 glomerulonephritis; aHUS, atypical haemolytic uremic syndrome; PSGN, post-streptococcal glomerulonephritis; HAE, hereditary angioedema, AAE, acquired angioedema*.

Anti-C1q is mainly analyzed by EIA. To avoid false-positive results in anti-C1q antibody analysis, it has been recommended that either only the collagenous part of C1q should be used as the antigen, or that high-salt buffer is used to abolish binding between the globular part of C1q and the Fc region of IgG in immune complexes (41). There are several commercially available assays to detect anti-C1q. In addition, Western blot analysis of the separated C1q A, B, and C chains (after reduction) has been used to show that anti-C1q antibodies have different binding specificities in SLE than in (HUVS) (42, 59).

### C3 nephritic factors and other convertase autoantibodies

C3NeF are autoantibodies that bind to and stabilize the AP C3 convertase (C3bBb) to prevent its extrinsic or intrinsic decay (60), prolonging the half-life of the convertase and resulting in increased consumption of C3. C3NeF are frequent in patients with (C3G; hence the name “nephritic factor”), where they are found in ~80% of DDD cases and 40-50% of patients with C3GN (61). C3NeF can also be found in other conditions such as acquired partial lipodystrophy and an increased susceptibility to meningococcal infections secondary to persistently low C3 concentrations resulting from C3 consumption (62, 63).

Given the heterogeneity of C3NeF with regard to binding specificities and convertase-stabilizing effects, it is considered necessary to use more than one method for analysis (64, 65). Also, different methods are needed to demonstrate both the convertase-stabilizing capacity and the Ig nature of C3NeF. Most C3NeF have been shown to bind to the Bb part of the convertase (66). The heterogeneous nature of C3NeF was already described about 30 years ago in studies that showed differences regarding the dependency on properdin and the ability to activate the AP (67, 68). A recent study has also confirmed that some C3NeF are more efficient convertase stabilizers in the presence of properdin; these antibodies have been termed C5 nephritic factors (C5NeF) and shown to be associated with C5 consumption as well as low C3 (69).

C3NeF can be analyzed in several ways. The most widely used assays are (1) detection of fluid-phase C3 conversion after incubation of patient serum with normal serum at 37°C (70, 71) and (2) a simple haemolytic assay that utilizes unsensitized sheep erythrocytes (72). However, these functional assays are non-specific and do not establish that the C3NeF effects are caused by an antibody. Therefore, other types of EIA methods have been developed in which an AP C3 convertase is deposited in a microtiter plate (64, 73). Standardization of C3NeF analysis is as yet lacking, but recently an external quality assessment (EQA) program that includes C3NeF analysis has become available as the result of an international initiative to standardize complement analyses (74).

Rarely, patients will present autoantibodies against the CP convertase (C4b2a); these are known as C4 nephritic factors (C4NeF). Like C3NeF in the AP, these autoantibodies stabilize the CP convertase, leading to persistent C3 consumption. Also, like C3NeF, they are principally found in association with glomerulonephritis (75, 76). C4NeF can be identified via haemolytic assays in which the CP convertase is stabilized by C4NeF on sensitized sheep erythrocytes (75).

### Anti-C1 inhibitor autoantibodies

Some patients with AAE have autoantibodies to C1-INH (anti-C1-INH), which can be of any Ig class. In AAE patients with monoclonal gammopathy, the Ig isotype of the M component is often identical to the anti-C1-INH isotype (77). Anti-C1-INH can increase the consumption of C1-INH or block its function, and AAE patients with anti-C1INH can benefit from treatment with B cell-inhibiting therapy (78). Although anti-C1-INH antibodies are much more common in AAE, they occur in a small fraction of patients with SLE, and anti-C1-INH IgM has also been reported in patients with HAE (79, 80). Anti-C1-INH antibodies are analyzed by EIA. Anti-C1-INH IgG, IgA, and IgM should all be determined, an unusual requirement in the context of autoantibody analysis. The assays may either detect the binding of anti-C1-INH to C1-INH or determine the capacity of the anti-C1-INH to block C1-INH function (81). Various in-house methods are used, since commercial methods are not available.

### Anti-factor H autoantibodies

Autoantibodies against factor H are detected in 6–10% of patients with aHUS and are also found in some patients with C3G. In aHUS, anti- factor H antibodies are mainly specific for the C-terminal part of factor H, and thus they block the ability of factor H to bind negatively charged carbohydrate residues on autologous cells. In C3G, anti- factor H may be directed toward other parts of factor H and may also be monoclonal or light chain-restricted (82–84). In aHUS, positivity for anti-factor H is strongly associated with a deletion of the genes for complement FHR-1/3 proteins, a deletion that is also common in the general population. aHUS with anti- factor H is considered a separate subgroup of aHUS for which the term “deficiency of CFHR plasma proteins and factor H autoantibody positive haemolytic uremic syndrome” (DEAP-HUS) has been proposed (85). Patients with anti- factor H -related aHUS will in most cases benefit from plasmapheresis and immunosuppressive treatment, unlike patients without anti- factor H, in whom C5-blocking therapy is mandatory (86). This difference implies that anti- factor H is an important diagnostic marker that needs to be analyzed rapidly.

Anti- factor H antibodies are analyzed by EIA. A multi-laboratory comparison of various in-house methods in 2014 established a recommended standard method, and a standard serum for calibration of arbitrary units is available (87). Commercial analysis kits are also available for anti- factor H determination.

### Other complement autoantibodies

Anti-complement autoantibodies with several other specificities have been described in association with various diseases. For example, autoantibodies against factor B have been detected in C3 glomerulopathy and membranoproliferative glomerulonephritis (MPGN) (88, 89), antibodies against factor I in aHUS (90), antibodies against MBL in rheumatoid arthritis (91), and antibodies against ficolins in SLE (92, 93). However, the clinical significance of most of these antibodies is not clearly defined, and their analysis has not been adopted in regular clinical practice. Autoantibodies with specificity for C3 and C4 fragments, termed immunoconglutinins, are found in different inflammatory conditions and in SLE, where they can influence C3-mediated functions (39, 94). More recently, antibodies recognizing different C3 fragments were investigated in patients with lupus nephritis and were found to be more common in patients with more severe disease (95).

## Conclusions

In summary, complement biomarkers can be used to follow the activity of a huge number of diseases and disorders in individual patients if they are compared with baseline values of the same individual. However, independent evaluations of the complement status in individuals without previous analyses are applicable on a relatively limited number of conditions due to low sensitivity and specificity of the existing assays and to preanalytical problems. Example profiles for a typical case of each condition are presented in Table 3. It is likely that introduction of new complement modulatory drugs with novel indications will increase the demand for complement monitoring in the near future.

## Authors contributions

KE, LS, and BN wrote the article. CM prepared the figures. BP and KS edited, and all authors approved the final manuscript.

### Conflict of interest statement

The authors declare that the research was conducted in the absence of any commercial or financial relationships that could be construed as a potential conflict of interest.
